# From biomarkers to a clue of biology: a computation-aided perspective of immune gene expression profiles in human type 1 diabetes

**DOI:** 10.3389/fimmu.2012.00320

**Published:** 2012-10-25

**Authors:** Dongmei Han, Xiaodong Cai, Ji Wen, Norma S. Kenyon, Zhibin Chen

**Affiliations:** ^1^Diabetes Research Institute, University of Miami Miller School of MedicineMiami, FL, USA; ^2^Department of Electrical and Computer Engineering, University of MiamiCoral Gables, FL, USA; ^3^Department of Medicine, University of Miami Miller School of MedicineMiami, FL, USA; ^4^Department of Surgery, University of Miami Miller School of MedicineMiami, FL, USA; ^5^Department of Microbiology and Immunology, University of Miami Miller School of MedicineMiami, FL, USA

**Keywords:** autoimmunity, computation, diabetes, human, gene expression

## Abstract

Dysregulated expression of key immune genes may cause breakdown of immunological tolerance and development of autoimmune disorders such as type 1 diabetes (T1D). General immune insufficiencies have also been implicated as a trigger of autoimmunity, due to their potential impact on immune homeostasis. Recent studies have detected evidence of systemic reduction in immune gene expression in long-term diabetic patients but the changes were not present before or at T1D onset. The changes could not be merely correlated with alteration in metabolic parameters. The studies also identified a dynamic expression pattern of several well-known as well as little-studied, immune-related genes during the course of T1D. An intriguing “ratio profile” of immune regulatory genes, such as CTLA4 and members of the S100 family, versus “baseline” immune genes, such as CD3G, prompted us to further examine immune gene expression relationships for a set of molecules representing T cells, B cells, and myeloid cells. No evidence was found to suggest an overall breach of tolerance equilibrium in T1D. Perplexingly, patients with long-term T1D presented a gene expression profile that was surprisingly more coordinated in analyses of “networking” relationship. Computational analyses of the “ratio profiles” or “relationship profiles” of immune gene expression might provide a clue for further studies of immunobiology in human T1D and other autoimmune diseases, as to how the profiles may be related to the pathogenic cause of the disease, to the effect of the diseases on immune homeostasis, or to an immunological process associated with the course of the diseases but is neither a direct cause nor a direct effect of the diseases.

## IMMUNOBIOLOGY AND IMMUNE BIOMARKERS IN T1D

It is generally accepted that type 1 diabetes (T1D) develops as a result of breakdown in immunological tolerance induction mechanisms. The contribution of various tolerance mechanisms has been clearly delineated with the aid of animal models of T1D. It is postulated that T1D and other autoimmune diseases are likely caused by specific breaches in a network of immunological tolerance mechanisms (Mathis and Benoist, 2010). However, a general defect at a system level has also been implicated by clinical and experimental evidence (Gallegos and Bevan, 2004; King et al., 2004; Schuetz et al., 2010). Indeed, studies with animal models suggested that autoimmune diabetes could be caused by either specific defects in immune dysregulation (for review, see Lehuen et al., 2010), or a general immune insufficiency (lymphopenia; King et al., 2004). However, it is difficult to pinpoint the breakdown of immunological tolerance in human patients. In common cases of human T1D, the status of the immune system remains a vaguely delineated framework that awaits characterization at molecular and cellular levels.

In the mean time, studies of biomarkers that are associated with T1D are expected to provide important tools for clinical management of the disease. Biomarkers can be derived from a broad spectrum of factors (Purohit and She, 2008), for example, T1D-associated genetic loci, antibodies against endocrine pancreatic tissues and products, potentially pathogenic or protective cytokines, and expression profiles of immunological or metabolic genes. The best examples are perhaps the predicative values of anti-insulin and anti-islet-cell antibodies for T1D development (Eisenbarth et al., 1998). Another well-recognized example is the measurement of hemoglobin A1c (HbA1c), which is commonly used for clinical diabetes management. Since immunological processes are believed to play critical roles in all stages of T1D development, immune gene expression profiles can potentially be used as biomarkers for staging T1D development as well as gauging the impact of the disease.

Microarray gene expression profiles of peripheral blood provided new insights into pathogenesis of T1D (Reynier et al., 2010). Such studies could reveal differences in immune responsiveness between patients with T1D and healthy controls, and may identify changes in gene expression that associate with progression of T1D. Studies of the peripheral immune system of new-onset T1D patients have shown significantly higher levels of IL-1α, IFN-γ, and TNF-α as compared to normal controls (Hussain et al., 1996; Kallmann et al., 1997). Patients with childhood-onset T1D show abnormal monocyte gene expression levels with an altered gene expression network, implicating monocyte abnormalities in susceptibility to diabetes (Beyan et al., 2010). Two distinct gene expression profiles have been reported in monocytes from peripheral blood: a proinflammatory profile mainly associated to adult-onset and a chemotaxis, adhesion, and metabolism profile mainly associated to juvenile-onset diabetes (Padmos et al., 2008). Recently, a study showed that healthy first-degree relatives of patients with T1D exhibited significant differences in expression pattern of genes involved in the regulation of innate immune responses such as TLR signaling and CCR3 signaling in eosinophils, costimulation, and cytokine responses mediated by CD137, CD40, and CD28 signaling and IL-1 proinflammatory pathway, as compared to healthy controls (Stechova et al., 2011).

One of the major subsets in the adoptive immune system, B lymphocytes, have been implicated to play a role in T1D development and disease progression (Serreze et al., 1998; Silveira and Grey, 2006), but the actual role that B cells play remains to be elucidated (Serreze et al., 1998; Wong et al., 2004). Several studies from animal models of autoimmune disease have shown that B cells can serve as antigen-presenting cells and prime β cell-specific T cells (Falcone et al., 1998; Serreze et al., 1998; Bouaziz et al., 2007). Data from a recent clinical trial demonstrated that treatment of new-onset T1D patients with anti-CD20 (Rituxan) preserved residual insulin secretion for at least 1 year (O’Neill et al., 2009; Pescovitz et al., 2009). B cells may also contribute to immune responses through the secretion of effector cytokines (Harris et al., 2005a,b; Duddy et al., 2007). Gene expression levels of two key B cell markers, activation-induced cytidine deaminase and immunoglobulin G gamma chain, were found significantly lower in long-term T1D patients as compared to healthy controls or new-onset patients (Han et al., 2011). The cytotoxic lymphocyte gene products granzyme B (GB), perforin, and fas ligand (FasL) have been shown to play a vital part in the T1D development (Kreuwel et al., 1999; Moriwaki et al., 1999; Kreuwel and Sherman, 2001; Yoon and Jun, 2001). mRNA levels of perforin and FasL genes were found significantly lower in patients with long-term T1D as compared to healthy controls (Giordano et al., 1995; Han et al., 2005).

It can perhaps be agreed that there are a plethora of examples of conflicting reports in immune-related changes in the course of T1D. For example, conflicting evidence exists on whether an active Th1-like immune response destroys beta cells, followed by presentation of autoantigens during the prediabetic phase (Karlsson Faresjo et al., 2004; Hedman et al., 2006). Th17 cells have been shown to play a critical role in the induction of autoimmune tissue injury and inflammation, and might be involved in exacerbation of diabetes, but its role in T1D remains to be clarified (Cooke, 2006; Bending et al., 2009; Emamaullee et al., 2009; Martin-Orozco et al., 2009; Honkanen et al., 2010). Increased levels of IL-17 secreting T cells were found in children with new-onset T1D (Marwaha et al., 2010). There are also conflicting reports about the levels of cytokine secretion and cytokine mRNA expression in at-risk, new-onset, and long-term T1D patients (Halminen et al., 2001; Nicoletti et al., 2002). These discrepancies might be contributed by different stages of the disease even within the same group, or sampling variations.

Another example is CTLA4, an immunoregulatory molecule that plays a key role in negatively regulating T cell responses (for review, see Teft et al., 2006). Genetic variations in the CTLA4 locus is associated with a number of autoimmune diseases (for review, see Kristiansen et al., 2000). Many groups studied potential dysregulation of CTLA4 expression in T1D. The mRNA expression level for CTLA4, as well as ICOS and GITR, was found lower in regulatory T (Treg) cells of children with newly diagnosed diabetes as compared to the healthy controls (Luczynski et al., 2009). CTLA4 protein expression was reported lower in the patients with diabetes (Haseda et al., 2011; Ryden et al., 2012) and with autoimmune thyroiditis as compared in controls (Kucharska et al., 2009). The expression of CTLA4 is influenced by genetic polymorphisms, although it remains debated how exactly each of CTLA4 polymorphisms impact human T1D genetics and how they contribute to CTLA4 expression variation (Ueda et al., 2003; Anjos et al., 2005). It should be noted that the reported changes in CTLA4 expression were subtle. A subtle reduction of CTLA4 could indeed impact T1D development (Chen et al., 2006), with mechanism remain to be elucidated. On the other hand, induction of CTLA4 is associated with tolerogenic effect of a therapeutic agent, the murine analog of anti-thymocyte globulin (ATG), in a transgenic mouse model of T1D (Lu et al., 2011). In experimental settings of CTLA4-deficiencies, i.e., “all-or-nothing” modeling, or anti-CTLA4-antibody-mediated blockade, it has been shown that CTLA4 acts on both Treg and effector T (Teff) cells (Wing et al., 2008; Peggs et al., 2009; Ise et al., 2010; Jain et al., 2010; Pruitt et al., 2011; Miska et al., 2012). However, in a recent study of antitumor immunity, Teff cells exhibited unique sensitivity to subtle reduction of CTLA4 (Miska et al., 2012).

In our studies to identify potential biomarkers in association with T1D progression, gene expression analyses were performed with quantitative RT-PCR for the mRNA levels of a set of immune-related genes using commercially tested primer and probe sets (Han et al., 2011, 2012). In this initial stage of studies, whole blood samples from at-risk, new-onset, and long-term T1D patients, as well as healthy controls were preserved for analyses after collection, without further processing or fractioning, to avoid any potential loss and changes caused by processing. It was found that IFN-γ, IL-4, and IL-10 mRNA levels were significantly higher in new-onset as compared to at-risk and long-term T1D patients (Han et al., 2011). The gene expression levels of most cytokines and effector molecules were suppressed in long-term T1D patients as compared to healthy controls (Han et al., 2011). Surprisingly, CTLA4 expression levels *per se* were not changed in the at-risk or new-onset stages, but reduced in long-term diabetic patients (Han et al., 2012). Unexpectedly, we found a significant difference between healthy controls and T1D groups in mRNA levels for “baseline” immune gene such as *CD3G* (representing T cells), *CD20* (representing B cells), and *CD11b* (representing myeloid cells). When CTLA4 expression was examined in reference to CD3G expression, as a ratio of CTLA4/CD3G, we detected a decreased ratio in the sample from at-risk and new-onset patients but an increased ratio in samples from long-term T1D patients. The distinct “ratio profiles” for various immune regulatory genes (Han et al., 2012) prompted us to further examine the relationship of immune gene expression in different stages of T1D.

## “RELATIONSHIP PROFILE” OF INNATE AND ADAPTIVE IMMUNE GENE EXPRESSION

It is thought that “cross-talk” between different subsets of immune cells is crucial for immunological tolerance (Lehuen et al., 2010; Suzuki et al., 2010), but it remains a challenge to study immune cell interaction in human patients. The gene expression values were obtained for T lymphocyte genes (CD3G and CTLA4), B lymphocyte genes (CD19 and CD20), and myeloid cell-related genes (CD11b, TLR9, and ARG1), as well as a subset of members of the *S100* family that has been implicated in immune regulation (Han et al., 2012). We probed the relationship of these values, by a computational approach, to gain a clue to the systemic and regulatory relationships among T-, B-, and myeloid cells. Whereas the well-established immunological relationships between CD3G and CTLA4, as well as CD19 and CD20, should validate the experimental and computational methods of gene expression relationship and network analyses, the inclusion of the *S100* family members in the expression analyses (Han et al., 2012) attests the approaches’ utility to explore novel relationships between a gene with a well-defined immunological function and another gene with a yet-to-be characterized role in immunity and tolerance.

**FIGURE 1:**
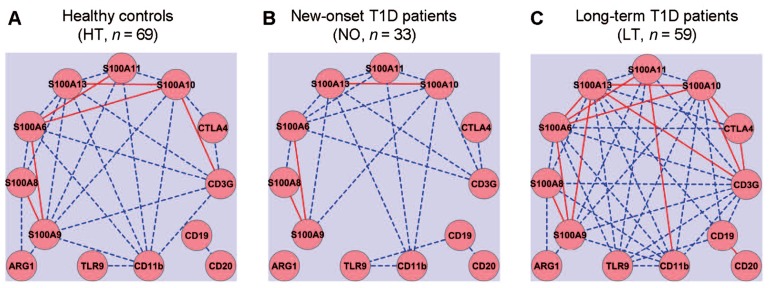
**Gene expression network analysis to examine potential coordination among innate and adaptive immune gene expression.** Quantitative RT-PCR was used to assess the levels of mRNA expression for thirteen innate (CD11b, TLR9, and S100 family) and adaptive (CD3, CTLA4, CD19, and CD20) immune genes. Gene expression relationships were analyzed for healthy controls **(A)** and compared to that of new-onset **(B)** and long-term T1D patients **(C)**. Solid red lines, absolute value of correlation coefficient ≥0.9; dashed blue lines, absolute value of correlation coefficient ≥0.8 but <0.9.

Gene expression networks were analyzed by calculating Pearson’s correlation coefficient among the expression values of each pair of genes, measured in a previously reported study (Han et al., 2012). Correlation between any two genes with an absolute coefficient value greater than 0.8 was presented in gene expression network graphs (**Figure 1**). A robust correlation between four pairs of genes was expected in the healthy samples, based on the established function of these genes: (1) *CTLA4* and *CD3G*; (2) *CD19* and *CD20*; (3) *CD11b* and *TLR9*; and (4) *S100A8* and *S100A9*. The anticipated relationships were designed to be a “positive” control for the computational method. Indeed, strong correlations of expression between all four pairs of genes were validated in the healthy control samples (**Figure 1A**). The correlation between the T cell pair and the B cell pair appeared to be relatively weak (<0.8) in the healthy group (**Figure 1A**). The sample size in the at-risk group (*n* = 19; Han et al., 2012) may be too small to estimate correlation coefficients reliably. Therefore, the at-risk group was not included in this computational analysis. In the healthy control group, one sample was discarded from this analysis because it contained statistical outliers (*n* = 69; Han et al., 2012).

The gene expression network was constructed using a standard formula for Pearson’s correlation coefficient. The computation analysis with this formula does not predict how the correlation coefficients of pairs of genes in one subject group would change when the average expression levels of a set of genes are all reduced, for example, whether the absolute values of the correlation coefficients would increase, decrease, or remain similar. Intuitively, we expected that the onset and duration of T1D might be associated with a disruption of a gene expression correlation network that exists in the healthy controls. Contrary to our expectation, we did not detect an apparent disruption of the gene expression correlations in the samples from the new-onset diabetes group (*n* = 33; **Figure 1B**). Instead, it was clear that the basic correlation pattern was preserved in the new-onset group as compared to the HT controls. Surprisingly, when compared to healthy controls (**Figure 1A**), the gene expression correlation network was much strengthened overall in long-term diabetic patients (*n* = 59; **Figure 1C**).

As expected, the expression of several *S100* genes correlated with the mRNA level of CD11b, an indicator of myeloid cells. Novel relationships of gene expressions were identified in the samples from the healthy group between *S100 *family genes and T cell genes (*CTLA4* and *CD3G*), but not with B cell genes (*CD20* and *CD19*). Surprisingly, although healthy controls and new-onset T1D patients exhibited a similar pattern of gene expression relationships, more correlative relationships of gene expression were identified in the long-term diabetic group than in healthy controls, such that the expression of *CD3G*, *CD11b*, *S100A6*, *S100A9,* *S100A10*, *S100A11*, or *S100A13* correlated with a majority of the other genes analyzed in the study, regardless of their primary functional denotation in T-, B-, or myeloid cells. Distinct gene expression relationships also emerged in the samples from the new-onset and long-term T1D groups between the B cell gene CD19 and TLR9, or between CD19 and members of S100 gene family (**Figures 1B,C**).

**FIGURE 2:**
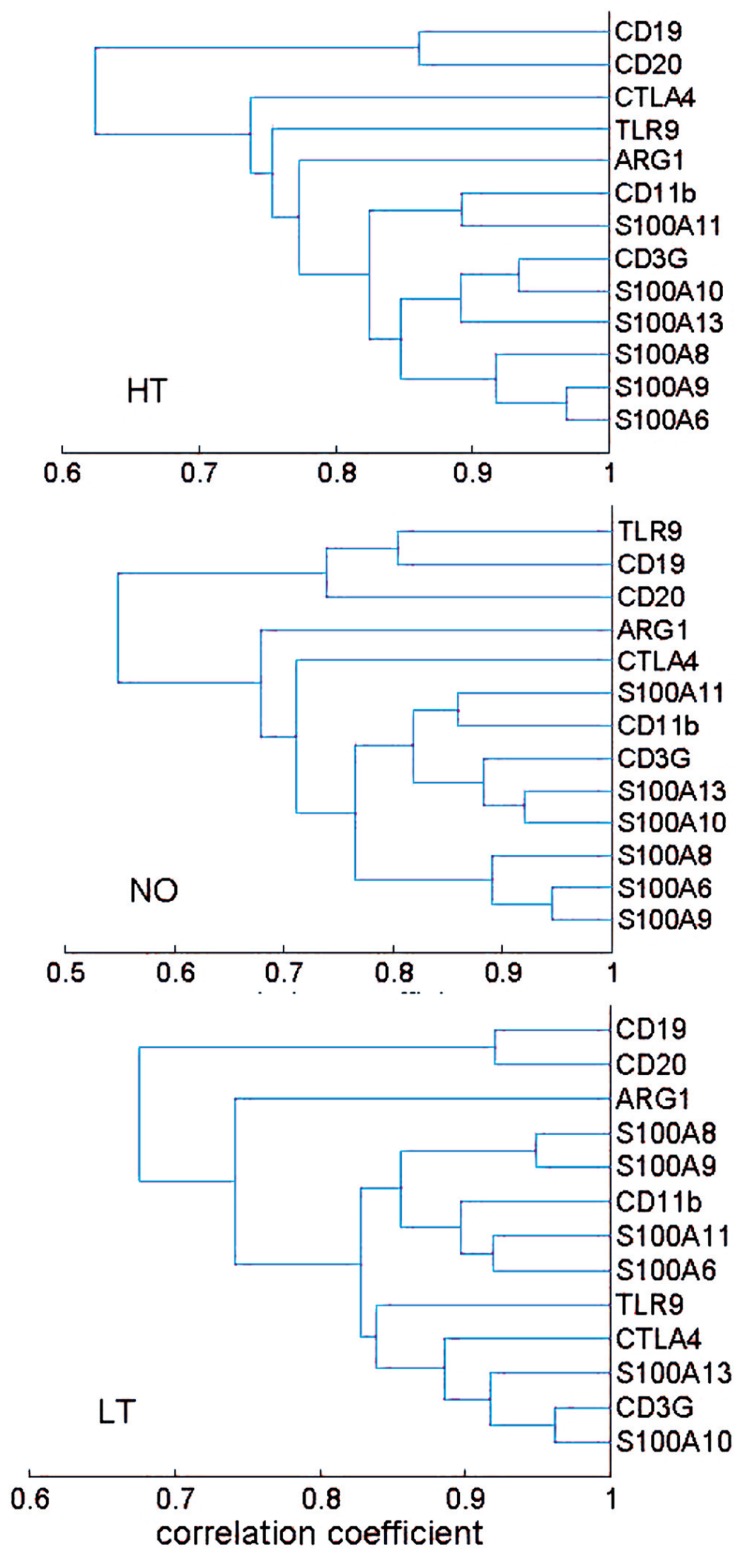
**Hierarchical clustering analysis of expression relationship among innate and adaptive immune genes.** A dendrogram was presented based on correlation coefficient of gene expression levels. The HT, NO, and LT groups exhibited a similar pattern of gene expression correlation. Most of the 13 genes appeared in two clusters: a B cell cluster and a cluster consisting of both T cell and myeloid cell genes. The sample size in the AT group (*n* = 19) may not be sufficient for the clustering technique.

The correlation coefficients were further used as a distance metric in a hierarchical clustering analysis that yielded a dendrogram, a commonly used method for depiction of relationship analyses. The healthy, new-onset and long-term T1D groups generally exhibited similar clusters (**Figure 2**). Thus, the results from the hierarchical clustering analyses support the finding from network analyses, suggesting that the expression relationships of the selected immune genes were well-preserved during progression of T1D.

## CROSS-GROUP ANALYSIS OF GENE–GENE INTERACTIONS

For experimental biologists to identify gene expression relationship, the most straightforward method is perhaps to simply analyze the ratio of gene expression levels for genes that are known to be related (Han et al., 2012), as described above. Statistical analyses of correlation coefficients, as described in **Figures 1** and 2, are also intuitively comprehensible and desirable by experimental biologists to detect potential relationship. However, other types of interactive relationships could exist and may necessitate more sophisticated computation methods. In genetic association studies, interactions of several genetic loci were found, in addition to the other well-established loci such as HLA, to be associated with T1D (Cordell et al., 1995; Ide et al., 2004). Gene–gene interactions are believed to be an important factor that may account for missing heritability for T1D (Pociot et al., 2010) and other complex human diseases (Manolio et al., 2009). We therefore employed a computational approach to identify gene–gene interactions that were associated with a T1D group vis-à-vis the healthy controls, using the gene expression values.

When we compared a T1D group with the healthy group, we used an indicator variable *Y* to indicate whether a subject is from the T1D group (*Y *= 1) or the healthy group (*Y *= 0), and then employed the following logistic regression model to investigate the effect of gene expression and the age on the status of the subject,

$$\log\left(\frac{P(Y=1)}{P(Y=0)}\right)=\beta_{0}+\overset{13}{\underset{i=1}{\Sigma }}X_{i}\beta_{i}+\overset{12}{\underset{i=1}{\Sigma }}\overset{13}{\underset{j=i+1}{\Sigma }}X_{i}X_{j}\,\beta_{ij}+age\,\times \beta_{a},$$

where *P*(*Y *= 1) or *P*(*Y *= 0) is the probability that the subject belongs to the T1D or healthy group, *X*_*i*_ is the expression level of gene *i*, and β_*i*_, β_*ij*_, β_*a*_ are regression coefficients to be determined. This logistic regression model is similar to the one used to detect gene–gene interactions in genome-wide association analysis (Cordell, 2009), except the variables here are gene expression values and the age instead of genotypes. Since the number of variables in the model is greater than the number of samples available, the traditional maximum likelihood approach cannot be used to determine the regression coefficients. We thus employed a method named elastic net (Zou and Trevor, 2005), which is capable of selecting relevant variables from a set of a large number of variables. The elastic net was originally used to predict if a leukemia patient has type 1 or 2 leukemia using the expression levels of 7129 genes (Zou and Trevor, 2005). Specifically, the elastic net determined the values of regression coefficients by maximizing the following penalized likelihood function,

$$L(y|\beta )-\frac{\lambda (1-\alpha )}{2}(\overset{13}{\underset{i=1}{\Sigma }}\beta_{i}^{2}+\overset{12}{\underset{i=1}{\Sigma }}\overset{13}{\underset{j=i+1}{\Sigma }}\beta_{ij}^{2})-\lambda \alpha (\overset{13}{\underset{i=1}{\Sigma }}|\beta i|+\overset{12}{\underset{i=1}{\Sigma }}\overset{13}{\underset{j=i+1}{\Sigma }}|\beta_{ij}|),$$

where *L*(*y*|β) is the log likelihood function of the data with β standing for all regression coefficients, α (0 < α ≤ 1) and λ (>0) are two constants that could be determined with cross-validation. We used an efficient program named glmnet (Friedman et al., 2010) that implemented the elastic net to fit the data to the logistic regression model. The constants α and λ were chosen from leave-one-out cross-validation that yielded the smallest deviance. The standard errors of non-zero coefficients obtained from glmnet were then calculated from the sandwich formula for the penalized regression models (Fan and Li, 2001).

In the analysis for LT versus HT group, cross-validation yielded α = 0.71 and λ = 0.01393. Hosmer–Lemeshow test for the goodness of fit of the model gave a *p*-value of 0.567 which implies that the data fitted the model properly. The area under the receiver operating characteristic (ROC) curve is 0.831, which also indicates that the data fitted the model well. The regression coefficients obtained from the glmnet were presented in **Table 1**. Three pair-wise gene interactions were identified associating with long-term T1D vis-à-vis healthy controls: *ARG1* versus *S100A6*, *ARG1* versus *S100A9*, and *CD3G* versus *CD20*, since their corresponding regression coefficient is ≠0 with a statistical significance <0.05. To illustrate the effect of gene-gene interactions, let us denote the probability of a subject belonging to the long-term T1D group as *p* and correspondingly the probability of subject being in the healthy group as 1 - *p*. The odds for the subject being associated with the long-term T1D group is then *o* = *p*/(1 - *p*)*.* Taking the analysis of CD3G versus CD20 for illustration, the regression coefficient is 0.21 (**Table 1**).

**Table 1:** Elastic net regression analyses of gene–gene interaction between the HT and LT-T1D groups.

	ARG1	CD19	CD3G	CTLA4	CD11b	CD20	S100A10	S100A11	S100A13	S100A6	S100A8	S100A9	TLR9
ARG1	0	0	0.07	0	0	0	0	0	0.03	0.22*	0.01	-0.21*	0
CD19		0	0	0	0	0	0	0	0	0	0	0	0
CD3G			0	0	0	0.21*	0	0	0.06	0	0	0	0
CTLA4				0	0	0	0	0	0.03	0	0	0	0
CD11b					0	0	0.02	0	0	0	0	0	0
CD20						0	0	0	0.04	0	0	-0.15	0
S100A10							0	0	-0.05	0	0	0	0.11
S100A11								0	0	0	0	0	0
S100A13									-0.62**	0.09	0	-0.11	0.01
S100A6										0	0	0	0
S100A8											0	0	0
S100A9												0	0
TLR9													0

*For a cross-group analysis of gene expression and interaction, gene expression data were fitted to a penalized logistic regression model named elastic net, according to an algorithm described in the text. Numbers in the table are the regression coefficients obtained from the analysis for healthy controls (HT) versus the long-term (LT)T1D group.*

* p < 0.05;

** p < 0.0005.This approach of multiple penalized regression did not detect any gene–gene interaction between HT versus AT, or HT versus NO groups.

In a hypothesized situation where the product of expression levels of CD3G and CD20 is increased by onefold but all other expression levels do not change, the odds of the subject being associated with long-term T1D is denoted in this situation as *o*_1_ and the odds ratio is defined as *o*_1_/o. The odds ratio *o*_1_/*o* = *e*^0.21^ = 1.23 implies that the relative likelihood of association with the long-term T1D group is increased by 1.23 times if the product of expression levels of CD3G and CD20 is increased by onefold under this hypothesized situation. Of course, this kind of hypothetical situation unlikely occurs, because (1) if the product of expression levels of CD3G and CD20 changes, most likely expression levels of CD3G and CD20 themselves change too, and (2) as we have seen expression levels of some genes are highly correlated; meaning, if expression levels of CD3G and CD20 change, expression levels of some other genes may change too. Therefore, the number does not really specify exact relative risk, but rather suggests that a characteristic CD3G and CD20 interaction indicates a biased association with the long-term T1D versus healthy condition. The immunological significance of this finding, as to how it is related to T1D, remains to be tested. It might suggest, although purely speculative at this point, an altered T–B collaboration in an established diabetes condition. Collaboration of T- and B-lymphocytes is critical for a productive immune response and its regulation.

Notably, this penalized regression model identified only one main effect of single genes between long-term T1D patients and healthy individuals (*S100A13*, *p* < 0.0005; **Table 1**), although both Student’s *t* test and linear regression analyses involving single genes and age indicated significant reduction of expression in 10 of the 13 genes (Han et al., 2012). This was due to the fact that the correlations among genes were high and the multiple penalized regression analysis only picked out the gene whose difference between the two groups was most significant. This approach did not identify any gene–gene interaction in cross-group analyses between HT versus AT, or HT versus NO.

## SUMMARY

A large on-going effort has been devoted by many groups to identify biomarkers for T1D and other autoimmune diseases. Undoubtedly, those biomarkers, including differential gene expression profiles, will be instrumental to improve the clinical management of the diseases. For some autoimmune diseases, T1D as a well-known example, even though diagnosis criteria are straightforward, biomarkers may prove to be a great aid in staging the disease as well as predicting disease development before and after onset. Given the immune origin of an autoimmune disease, immune gene expression profiles are logical candidates of biomarkers. Such profiles may or may not necessarily be directly related to the disease cause or effect, but regardless may be useful indicators for the status of the immune system, the “immunological wellness,” during the disease progression (Han et al., 2012).

Successful prevention or cure of T1D and other autoimmune diseases will require a better understanding of the mechanisms of the diseases. In this regard, substantial progress has been made. Multiple genetic risks and environmental factors are implicated in the immunological tolerance breakdown that leads to T1D. Rare cases of T1D caused by Mendelian genetic mutations at either the *AIRE* or *FOXP3* locus highlight the critical role of central tolerance by thymic deletion or peripheral tolerance by regulatory T lymphocytes, respectively (Mathis and Benoist, 2010). On the other hand, a surprisingly limited spectrum of autoimmune damage in Aire/Foxp3 double-deficient mice suggests robustness of an immunological tolerance framework reinforced by a network of known and unknown elements (Chen et al., 2005). Clinical observations and experimental studies suggest that a general immune insufficiency may cause autoimmune diseases (Gallegos and Bevan, 2004; King et al., 2004; Schuetz et al., 2010). For a T1D population consisting of common cases that are not caused by monoallelic genetic mutations, however, the overall immunological status during T1D initiation and progression remains a challenge to characterize, due to a lack of experimental approaches for a system level assessment. A daunting task remains to study the immunobiology and pathophysiology behind human T1D and other autoimmune diseases, due to obvious clinical and logistic limitations and ethical concerns.

Perhaps, it is fair to critique that the computational analyses would be difficult to interpret in the absence of experimental evidence, and therefore may not be suitable for an experimental journal unless experimental evidence is provided. Currently available experimental biology approaches are not handy yet to definitively tackle human immunology at a system level in a complex disease setting such as T1D. Subtle and specific imbalances in innate and adaptive immune regulation, e.g., CTLA4/CD3 ratios, could be detected and may be associated with the T1D development. Systemic perturbations might occur, apparently not as a cause of T1D but as a part associated with the T1D course (Han et al., 2012). Of course, the computational analysis of the gene expression relationships could be regarded as just another approach by another “blind man” to the disease “elephant.” However, we argue that it may provide a clue to the biology behind the disease, suggesting that T1D pathogenesis in humans is not due to a gross encroachment of the integrity of immune regulatory network, nor to a general insufficiency in the immune system. For a reason(s) yet to be determined, a strengthened correlation of immune gene expression was associated with T1D progression. It remains to be understood whether and how the altered relationships may impact immune function and immune tolerance induction. A tightened immune regulatory network might facilitate tolerance induction. On the other hand, an increased rigidity of a network might also affect its responsiveness. In that vein, the general insufficiency of immune gene expression in long-term diabetes, together with a tightened network of immune gene expression, might pose a secondary risk of suboptimal immunity, and a perturbation in the homeostasis of the immune system.

## Conflict of Interest Statement

The authors declare that the research was conducted in the absence of any commercial or financial relationships that could be construed as a potential conflict of interest.
